# Sublethal Effects of Pyridaben on the Predatory Function of *Neoseiulus womersleyi*

**DOI:** 10.3390/insects15090647

**Published:** 2024-08-28

**Authors:** Cancan Song, Chengcheng Li, Juan Wei, Hualan Zeng, Qunfang Yang, Surong Jiang, Chunxian Jiang, Qing Li

**Affiliations:** 1 College of Agronomy, Sichuan Agricultural University, Chengdu 611130, China; songcan1993@163.com (C.S.); gubei0512@gmail.com (C.L.); 18352887796@163.com (J.W.); lmk94811@163.com (Q.Y.); jiangsr2006@sohu.com (S.J.); chunxianjiang@126.com (C.J.); 2 Industrial Crop Research Institute, Sichuan Academy of Agricultural Sciences, Chengdu 610300, China; zhl0529@126.com

**Keywords:** pyridaben, *Neoseiulus womersleyi*, sublethal effect, predatory function, Niemann–Pick-type C2 (NPC2)

## Abstract

**Simple Summary:**

Pyridaben is an efficient, low-toxicity, broad-spectrum insecticide that impairs the predatory capabilities of predatory mites, but the specific mechanisms that affect the predatory functions remain underexplored. This study elucidated that pyridaben significantly diminished the predatory efficiency and searching behavior of the predatory mite *Neoseiulus womersleyi*. A gene linked to olfactive functions, *NwNPC2a*, was cloned from *N. womersleyi.* Post-treatment with pyridaben at LC_30_ and LC_50_ concentrations resulted in a substantial downregulation of *NwNPC2a* expression. Silencing *NwNPC2a* in *N. womersleyi* females led to significant reductions in predatory functions. The decrease in predatory performance at LC_30_ and LC_50_ concentrations was attributable to the suppression of *NwNPC2a* gene expression. Thus, the underlying molecular mechanism through which pyridaben compromises the predatory function of *N. womersleyi* likely involves the downregulation of *NwNPC2a* expression.

**Abstract:**

Pyridaben is a widely utilized, broad-spectrum contact acaricide, which has notable sublethal effects that impair the predatory capabilities of predatory mites, but the specific mechanisms that affect the predatory functions remain underexplored. When predatory mites hunt for prey, they may rely on Niemann–Pick-type C2 (NPC2) proteins to collect herbivore-induced plant volatiles (HIPVs) and other odor molecules to locate and pursue their prey. This study elucidated that pyridaben significantly diminished the predatory efficiency and searching behavior of the predatory mite *Neoseiulus womersleyi*. Key metrics, including predatory capacity (*a/Th*) and predation rate (*a*) on various developmental stages of *Tetranychus urticae*, were markedly reduced in treated mites compared to controls. The searching efficiency (*S*) also declined proportionally with the increased sublethal dose of pyridaben. A gene linked to olfactive functions, *NwNPC2a*, was cloned from *N. womersleyi*. Post-treatment with pyridaben at LC_30_ and LC_50_ concentrations resulted in a substantial downregulation of *NwNPC2a* expression by 60.15% and 58.63%, respectively. Silencing *NwNPC2a* in *N. womersleyi* females led to significant reductions in the attack rate (*a*), handling time (*Th*), predation efficiency (*a/Th*), and maximum predation rate (1/*Th*). The searching efficiency (*S*) was also lower than that of the control group, displaying a slight decline with the increasing prey density. The findings revealed that pyridaben exerted inhibitory effects on both the predatory function and searching efficiency of *N. womersleyi* populations. The decrease in predatory performance at LC_30_ and LC_50_ concentrations was attributable to the suppression of *NwNPC2a* gene expression. RNA interference (RNAi) studies corroborated that the *NwNPC2a* gene plays a critical role in the predation process of *N. womersleyi*. Thus, the underlying molecular mechanism through which pyridaben compromises the predatory function of *N. womersleyi* likely involves the downregulation of *NwNPC2a* expression.

## 1. Introduction

Pyridaben is an efficient and low-toxicity acaricide with strong contact action [1]. It is an acaricide with a wide spectrum of action against all plant-feeding mites [2], such as *Panonychus citri*, from eggs to adult mites [3]. However, with the extensive annual use of pyridaben, mite resistance to this acaricide is continuously increasing, necessitating the development of more efficient, safer, and more sustainable control technologies. Biological control, known for being environmentally friendly and not restricted by mite resistance, can be used in conjunction with acaricides to enhance control efficiency. For example, Iwassaki et al. [4] achieved a significant reduction in the damage levels of *Tetranychus urticae* by combining *Neoseiulus californicus* with pyridaben. Similarly, Jin et al. [5] reported effective control of *T. urticae* by combining *Phytoseiulus persimilis* with bifenthrin. Another study demonstrated that using two species of predatory mites in conjunction with etoxazole provided effective control of *T. urticae* [6]. Similarly, a study found that combining mineral oil emulsions with *Neoseiulus barkeri* provided good synergistic control of *P. citri* [7]. In the application of acaricides and predatory mites for controlling pest mites, acaricides not only directly kill mites but also unavoidably affect the behavior and physiology of naturally occurring or subsequently released predatory mites. These sublethal effects can reduce the reproductive capacity of predatory mites, diminish their prey-searching ability, and decrease their population size, potentially leading to pest mite resurgence and hindering pest control efforts [8,9].

Research on the sublethal effects of low doses of pyridaben on pest mites has primarily focused on life history traits (development time, fecundity, and longevity) and population parameters. Generally, these sublethal effects are inhibitory, limiting population growth by reducing the lifespan and reproductive capacity of adult females, affecting egg hatching, and prolonging the development period of the F1 generation. However, there are instances where sublethal effects can trigger “overcompensation effects” or “corrective overcompensation effects” in pest mite populations, leading to a stimulatory effect [10,11]. As pyridaben is a broad-spectrum acaricide with secondary insecticidal properties, it can also harm predatory mites, as well as some insect predators of plant-feeding mites. Consequently, recent research has shifted focus to the impact of sublethal effects of pyridaben on behavior, i.e., predation of pest mites by their natural enemies. Another study observed significant declines in the predation ability and searching efficiency of *N. californicus* after treatment with pyridaben at LC_10_ and LC_30_ concentrations [12]. Further research discovered that treatment with pyridaben at the LC_30_ concentration slightly reduced the predation of *N. barkeri* on *T. urticae* and significantly induced the activities of glutathione S-transferases (GSTs) and carboxylesterases (CarEs), while inhibiting multi-function oxidases (MFOs) [13].

Few researchers have deeply analyzed the mechanisms by which the sublethal effects of pyridaben impact the predatory functions of predatory mites. Plants generate herbivore-induced plant volatiles (HIPVs) when damaged by pests, which have been widely studied in plant pest resistance strategies. *N. womersleyi* may locate prey through HIPVs produced after *T. urticae* feeding, as demonstrated in attraction experiments with common predatory mites, such as *Neoseiulus cucumeris* and *P. persimilis* [14,15,16,17,18]. Due to the lack of eyes and antennae, *N. womersleyi* uses its chelicerae and legs to continuously tap and perform olfactive functions to locate prey. The physiological functions of Niemann–Pick-type C2 (NPC2) proteins in vertebrates, such as cholesterol and lipid binding and transport, are well known [19]. In contrast, studies on the functions of *NPC2* genes in insects and mites are limited. Some *NPC2* genes were highly expressed, specifically in the first pair of feet [20,21]. *NPC2* genes may be involved in the collection and transport of HIPVs in predatory mites, thereby participating in chemical communication for locating hosts and effective foraging [22,23,24,25]. Hypotheses suggest that NPC2 proteins in insects may rely on the hemolymph pH in antennae to alter binding affinities for different odor molecules, although this remains unconfirmed. Alternatively, NPC2 proteins may undergo conformational changes induced by odor molecules to bind various odors [26].

Currently, the relationship between *NPC2* genes and HIPVs in predatory mites and their connection to predatory function and searching efficiency requires further in-depth research. This study focuses on *N. womersleyi*, a mite with strong control capabilities against various pest mites, and uses *T. urticae*, a severely damaging spider mite, as prey. By examining the sublethal effects of pyridaben on the *NPC2* gene, this research aims to elucidate the mechanisms affecting the predation responses of natural enemies, providing theoretical support for resolving conflicts in the use of acaricides and predatory mites in practical production. This study has practical significance for the selection of safe acaricides, rational pesticide application, and the protection of natural enemies.

## 2. Materials and Methods

### 2.1. Mite Source and Chemicals

*N. womersleyi* mites were collected in 2021 from soybean leaves (no pesticides were applied during this period) outside the Entomology Laboratory at Sichuan Agricultural University. Members of this study sequenced the ITS and CoI sequences of this species, BLASTed the sequences in NCBI, and constructed a phylogenetic tree, showing them clustering with *N. womersleyi* (GenBank: AB618061.1) with 100% confidence. Specimens of both male and female mites were also sent to Dr. Fang Xiaoduan at the Institute of Zoology, Guangdong Academy of Sciences, for morphological identification on slide mounts, confirming them as *N. womersleyi*. Therefore, this paper used the name *N. womersleyi* for this species. The mites were continuously reared on *T. urticae* infesting soybean leaves (Zhenong No. 8) using the leaf disc method in a controlled climate chamber at a temperature of 25 ± 1 °C, relative humidity of 75 ± 5%, and a photoperiod of 16 h light and 8 h dark. During this period, they were not exposed to any chemical agents.

The 97% pyridaben technical material was obtained from Xinyi Taison Chemical Co., Ltd. (Xuzhou, China), acetone (AR grade) from Sichuan Xilong Chemical Co., Ltd. (Chengdu, China), and Tween-80 (AR grade) from Shanghai Biyuntian Biotechnology Co., Ltd. (Shanghai, China).

### 2.2. Acute Toxicity Bioassay

The LC_30_ and LC_50_ concentrations of pyridaben on adult female *N. womersleyi* mites were estimated using the leaf residue method, following and modifying the approach of Kim and Yoo [27]. Based on preliminary experiments, pyridaben stock solutions (1000 μg/mL) were prepared with acetone and diluted with 0.05% Tween-80 aqueous solution into five concentrations (12.5, 25, 50, 100, and 200 μg/mL). A mixture of 0.05% Tween-80 and acetone was used as a blank control. Bioassays consisting of 5 concentrations of pyridaben and a blank control were replicated three times, with total sample size of 540 mites [28]. The treatment procedure involved cutting soybean leaves into 4.5 cm-diameter circles, spraying the back of the leaves with the solutions, and allowing them to air dry. The treated leaves were placed in the center of glass petri dishes lined with moist cotton. Healthy adult female *N. womersleyi* mites were transferred onto the leaves using a fine brush, with 30 mites per leaf. The Petri dishes were sealed with perforated plastic wrap and placed in an artificial climate chamber at 25 ± 1 °C, 75 ± 5% RH, and a 16 h L:8 h D photoperiod. Mortality was recorded after 24 h by gently touching the mites with a brush—mites that did not move were considered dead. Data were considered invalid if control mortality exceeded 10%.

### 2.3. Effects of LC_30_ and LC_50_ Concentrations of Pyridaben on the Predatory Function of Adult Female N. womersleyi

Adult female *N. womersleyi* mites of the same age, developed from eggs, were starved for 24 h on blank leaves treated with LC_30_ and LC_50_ concentrations of pyridaben. In six-well cell culture plates lined with smooth, moist cotton and filter paper, a blank soybean leaf was placed in each well. Different numbers (10, 15, 20, 25, and 30) of *T. urticae* mites at various stages were introduced onto the leaves. A single surviving adult female *N. womersleyi*, treated and starved at different concentrations, was placed in each well. After 24 h, the number of surviving and consumed *T. urticae* mites at each stage was recorded [29,30]. A mixture of distilled water and acetone without pyridaben was used as a control, with six replicates per density. To avoid inaccuracies in predation rates caused by *T. urticae* eggs, eggs laid by female *T. urticae* were removed multiple times during the 24 h predatory period.

### 2.4. Cloning and Sequence Analysis of the NPC2 Gene in N. womersleyi

#### 2.4.1. Total RNA Extraction and Reverse Transcription from Adult Female *N. womersleyi*

Sample collection: 150 healthy adult female *N. womersleyi* mites of the same developmental stage were collected into a 1.5 mL enzyme-free, sterile centrifuge tube, flash-frozen in liquid nitrogen, and stored at −80 °C. Total RNA was extracted using the FastPure Cell/Tissue Total RNA Isolation Kit (Vazyme, Nanjing, China), following the manufacturer’s protocol. Reverse transcription of the total RNA into cDNA was performed using the HiScript III First-Strand cDNA Synthesis Kit (+gDNA wiper; Vazyme, Nanjing, China), according to the manufacturer’s instructions.

#### 2.4.2. Cloning of the *NPC2* Gene

Primers for the *NPC2* gene were designed based on the sequence of the *NPC2-b* gene from *N. californicus* (GenBank: OQ927575.1) (Table 1). All primers used in this study were synthesized by Tsingke Biotechnology Co., Ltd. (Chengdu, China). PCR amplification was performed using the 2×Taq PCR MasterMix (with dye; Solarbio, Beijing, China). The PCR products were examined by 1% agarose gel electrophoresis, and the target bands were excised under UV light. The gel was purified using the FastPure Gel DNA Extraction Mini Kit (Vazyme, Nanjing, China). The purified products were ligated into the pMD™19-T vector using the Vector Cloning Kit (TaKaRa, Beijing, China) and transformed into DH5α competent cells (Vazyme, Nanjing, China). Positive clones were verified by colony PCR, and 500 μL of the verified bacterial culture was sequenced by Sangon Biotech Co., Ltd. (Shanghai, China). The remaining bacterial culture was used for plasmid extraction using the FastPure^®^ Plasmid Mini Kit (Vazyme, Nanjing, China), and the plasmid concentration was measured and stored at −20 °C for further use.

### 2.5. qPCR of NPC2 Gene in N. womersleyi

For RNA extraction, adult female *N. womersleyi* mites of the same age treated with LC_30_ and LC_50_ concentrations of pyridaben were collected. Untreated mites of the same age served as the control group. Each treatment had three biological replicates. Reverse transcription for qPCR was performed using the HiScript II Q RT SuperMix for qPCR (+gDNA wiper) kit (Vazyme, Nanjing, China). Before qPCR, the cDNA concentrations from the blank control, LC_30_, and LC_50_ treatment groups were diluted or adjusted to 200 ± 10 ng/μL using RNase-free ddH_2_O. qPCR primers were designed online using NCBI’s Primer-BLAST. The reference gene primers for *N. womersleyi β*-actin (ACTB) were based on the design by Ding et al. [31] (Table 2). The target band size and location were verified by PCR and agarose gel electrophoresis to ensure primer quality. qPCR was conducted using the ChamQ Universal SYBR qPCR Master Mix kit (Vazyme, Nanjing, China).

### 2.6. RNAi Study of NwNPC2a Gene in N. womersleyi

#### 2.6.1. dsRNA Synthesis

Based on the *NwNPC2a* sequence, primers with T7 promoter sequences were designed online using the dsRNA primer design tool (https://www.flyrnai.org/snapdragon (accessed on 20 September 2023)). The green fluorescent protein gene (GFP) from *Aequorea victoria* was used as a blank control (Table 3). PCR amplification of the dsRNA template for *N. womersleyi* was performed using the plasmid obtained in Section 2.4.2 as the template and the dsRNA primers with T7 promoter sequences (Table 3). The PCR product was verified by agarose gel electrophoresis, followed by gel extraction, ligation, transformation, sequencing, and plasmid extraction, as described in Section 2.4.2. The high-concentration plasmid was stored at −20 °C. PCR amplification was repeated using the dsRNA primers with T7 promoter sequences (Table 3) and the high-concentration plasmid as the template. The gel-extracted PCR product was purified and stored at −20 °C. dsRNA was synthesized in vitro using the T7 RNAi Transcription Kit (Vazyme, Nanjing, China) and purified by the magnetic bead method. The purified dsRNA was stored at −20 °C.

#### 2.6.2. dsRNA Feeding

For the feeding method for dsRNA interference [32,33], a solution of 25% sucrose dye was prepared by mixing 0.125 g of sucrose, 0.075 g of cochineal dye, and 500 μL of distilled water. A mixture of 20 μL of a 1000 ng/μL dsRNA solution and 2 μL of sucrose dye solution was prepared. Using a pipette, 0.5 μL of the mixture was applied to soybean leaves for feeding adult female mites that had been starved for 24 h. The feeding process continued until the solution was fully applied. Mites that fed on the sucrose dye solution exhibited an enlarged and plump appearance, with the intensity of the cochineal dye color in their bodies increasing proportionally to their consumption, observable through the transparent exoskeleton.

#### 2.6.3. Interference Efficiency Detection

For each treatment, 150 healthy female mites with a deep red coloration were selected and collected into centrifuge tubes. Total RNA was extracted, and qPCR was performed as described in Section 2.3. dsGFP-treated mites served as the blank control, with three biological replicates per treatment.

#### 2.6.4. Observation of Predation Ability Post-Interference

Thirty healthy adult female mites with deep red coloration were selected from each interference treatment, transferred to new blank soybean leaves, and starved for 24 h. Single mites were then placed in six-well cell culture plates containing five different densities of adult female *T. urticae.* After 24 h, the number of preys consumed was recorded. To avoid inaccuracies in predation rates due to *T. urticae* eggs, eggs laid by female *T. urticae* were removed multiple times during the 24 h predatory period. Each density was replicated six times.

### 2.7. Data Analysis

#### 2.7.1. Toxicity Estimation of Pyridaben on Adult Female *N. womersleyi*

The toxicity of pyridaben to adult female *N. womersleyi* was estimated using PoloPlus software for regression analysis. Toxicity regression equations were established, and sublethal concentrations were determined. The Chi-square fit test of the fitted virulence regression equation was carried out [34].

#### 2.7.2. Sublethal Effects of Pyridaben on the Predation Ability of Adult Female *N. womersleyi*

The predatory functional response curve was fitted using the Holling Type II disc equation [35], as follows:

$$N_{a}=\frac{aTN_{0}}{1+aT_{h}N_{0}}$$

where *N*_0_ is the initial prey density, *N_a_* is the number of preys consumed by the predator, *T* is the duration of the experiment (1 day in this study), *a* is the instantaneous attack rate of the predator, and *T_h_* is the handling time per prey. The maximum daily predation rate, *N_amax_* = 1/*T_h_*, and the predatory capacity is *a*/*T_h_*.

The disc equation was simplified to a linear equation: *y = kx + b*, using the reciprocal method, where *x* = 1/*N*_0_, y = 1/*N_a_*, *b* = *T_h_,* and *k* = 1/*a*. Linear regression analysis was performed using the least squares method to estimate parameters and obtain the disc equation.

During predation, the behavioral effect of the predator attacking the prey is termed the searching efficiency (*S*), calculated using the formula:

$$S=\frac{a}{1+aT_{h}N_{0}}$$

Experimental data were processed using SPSS 27.0, and SigmaPlot 12.5 software was used for plotting. The predation amount, preference, and searching efficiency of *N. womersleyi* for different stages of *T. urticae* were analyzed, along with changes in predatory function following sublethal pyridaben treatment.

#### 2.7.3. Bioinformatics Analysis

The obtained sequence of the *NwNPC2a* gene from *N. womersleyi* was analyzed for bioinformatics analysis. Similarity comparisons with *NPC2* gene sequences from other species were performed using NCBI BLAST (Table 4). The full-length sequence of the *NwNPC2a* gene was aligned in the GenBank database. A phylogenetic tree was constructed using the Neighbor-Joining method in MEGA 11.0 software, with bootstrap tests (1000 repetitions) for phylogenetic analysis.

#### 2.7.4. qPCR Analysis Following Sublethal Pyridaben Treatment

The relative expression of the *NwNPC2a* gene in female mites treated with LC_30_ and LC_50_ concentrations of pyridaben was analyzed using the 2^−ΔΔCt^ method, with three biological replicates. Significant differences were compared and plotted using one-way ANOVA in GraphPad Prism 9.5.1 software, with *p* < 0.05 indicating significance and *p* < 0.01 indicating high significance.

#### 2.7.5. Post-Interference qPCR Analysis

qPCR analysis post-interference was performed as described in Section 2.7.4.

#### 2.7.6. Analysis of Predation Ability Post-Interference

The analysis of predation ability post-interference was performed as described in Section 2.7.2.

## 3. Results

### 3.1. Toxicity Determination of Pyridaben on Adult Female N. womersleyi

The toxicity of pyridaben to adult female *N. womersleyi* is presented in Table 5. The median lethal concentration (LC_50_) of pyridaben for adult female *N. womersleyi* was determined to be 37.395 μg/mL, and the LC_30_ concentration was found to be 24.417 μg/mL.

### 3.2. Effects of LC_30_ and LC_50_ Concentrations of Pyridaben on the Predatory Function of N. womersleyi

#### 3.2.1. Effects of LC_30_ and LC_50_ Concentrations of Pyridaben on Predation

The effects of LC_30_ and LC_50_ concentrations of pyridaben on various predatory function parameters are shown in Table 6. The results indicate that both LC_30_ and LC_50_ treatments significantly reduced the predatory capacity (*a*/*T_h_*) and attack coefficient (*a*) of *N. womersleyi* on different stages of *T. urticae* compared to the control group. Additionally, as the concentration increased, both *a*/*T_h_* and *a* exhibited a general decreasing trend. However, under LC_30_ treatment, an increase in *a*/*T_h_* was observed for the predation on *T. urticae* larvae. Similarly, the maximum daily predation rate (1/*T_h_*) also showed an increase under LC_50_ treatment for eggs, LC_30_ treatment for larvae, and LC_30_ and LC_50_ treatments for nymphs, compared to the control group. These changes were attributed to a reduction in the handling time, *T_h_*, for *N. womersleyi* when preying on *T. urticae*.

#### 3.2.2. Effects of LC_30_ and LC_50_ Concentrations of Pyridaben on Searching Efficiency

**Figure 1:**
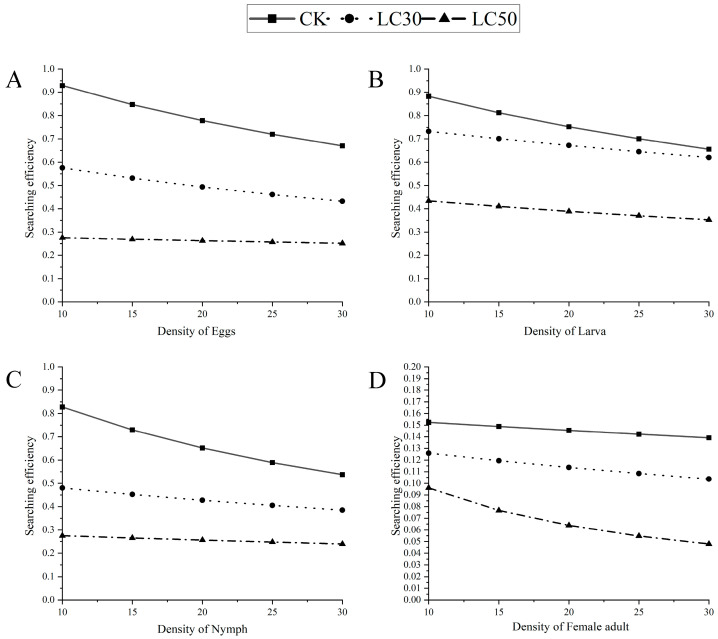
Relationship between the searching efficiency and density of *N. womersleyi* on the various mite states of *T. urticae* with sublethal effects of pyridaben. Graphs (**A**–**D**) correspond to different stages of the prey development: (**A**) egg stage of prey, (**B**) larva stage of prey, (**C**) nymph stage of prey, and (**D**) female adult stage of prey.

The searching efficiency equations for *N. womersleyi* on different stages of *T. urticae* under LC_30_ and LC_50_ treatments are shown in Table 7. Figure 1 illustrates the changes in searching efficiency compared to the control at different prey densities. The results indicate that the searching efficiency of *N. womersleyi* for all stages of *T. urticae* decreased with the increasing sublethal concentrations of pyridaben and was consistently lower than the control. The searching efficiency was also lower, displaying a slight decline with the increasing prey density.

### 3.3. Bioinformatics Analysis of NwNPC2a Gene

This study successfully cloned the olfactive protein gene *NwNPC2a* from *N. womersleyi* for the first time. The gene sequence was uploaded to the NCBI database and compared using NCBI BLAST. The sequence comparison revealed that *NwNPC2a* had the highest similarity (85.43%) with the *NPC2-b* gene of *N. californicus*. Analysis of the full-length sequence and encoded amino acid sequence of the *NwNPC2a* gene is shown in Table 8.

**Figure 2:**
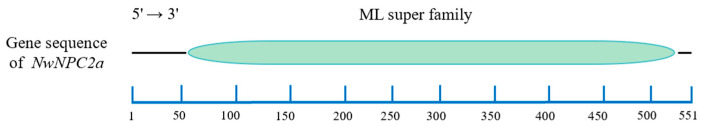
The conserved domains of gene *NwNPC2a*.

**Figure 3:**
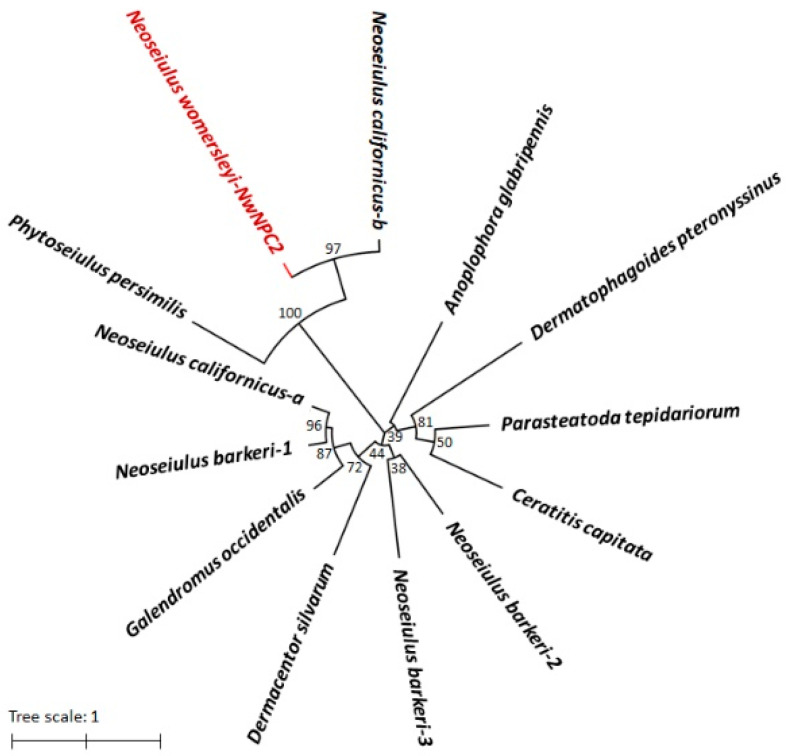
Phylogenetic tree of gene *NwNPC2a.* The GenBank accession numbers were as follows: *Neoseiulus californicus-a*, OQ927574.1; *Neoseiulus californicus-b*, OQ927575.1; *Phytoseiulus persimilis*, OQ413995.1; *Neoseiulus barkeri-1*, MT422741.1; *Metaseiulus occidentalis*, XM_018640306.1; *Dermacentor silvarum*, XM_037724110.2; *Dermatophagoides pteronyssinus*, XM_027342229.1; *Parasteatoda tepidariorum*, XM_016072263.2; *Ceratitis capitata*, XM_004536312.2; *Anoplophora glabripennis*, XM_018713713.1; *Neoseiulus barkeri-2*, MT422742.1; *Neoseiulus barkeri-3*, MT422743.1. The GenBank accession numbers starting with XM_ are based on the predicted sequence derived from the genome sequence.

The prediction of conserved domains in *NwNPC2a* revealed an ML superfamily conserved region (Figure 2), characteristic of *NPC2* genes, primarily involved in the specific binding and transport of lipid substances. This finding supports the functional consistency of *NPC2* proteins across different organisms. The high reliability of the cloned gene was further indicated by these conserved domains. A phylogenetic analysis of the *NwNPC2a* gene was conducted, revealing the highest homology with the *NPC2-b* gene of *N. californicus*, with a confidence level of 97% (Figure 3). The phylogenetic analysis results align with traditional taxonomic relationships.

### 3.4. qPCR Analysis of NPC2 Gene in N. womersleyi

**Figure 4:**
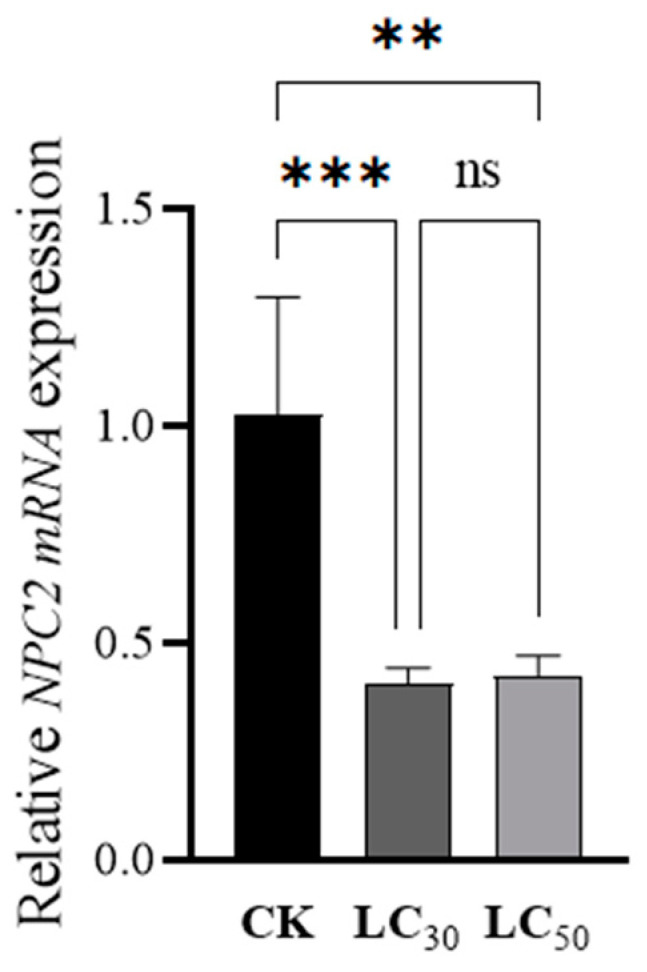
Relative expression levels of the *NwNPC2a* gene. Significant differences were compared and plotted using one-way ANOVA. *** Indicates *p* < 0.001, ** indicates *p* < 0.01, and ns indicates no significant difference.

qPCR analysis showed that the relative expression level of the *NwNPC2a* gene was significantly inhibited (*p* < 0.01) in *N. womersleyi* treated with LC_30_ and LC_50_ concentrations of pyridaben, with relative expression levels decreasing by 60.15% and 58.63%, respectively. There was no significant difference in relative expression levels between the two sublethal concentrations (*p* > 0.05; Figure 4).

### 3.5. Interference of NwNPC2a Gene

**Figure 5:**
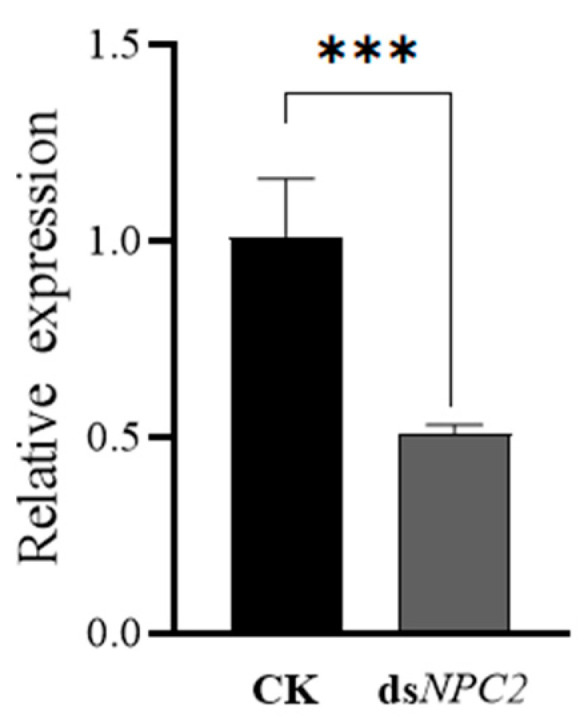
Relative expression levels of gene *NwNPC2a* after RNAi. Significant differences were compared and plotted using the independent samples *t*-test. *** Indicates *p* < 0.001.

After dsRNA interference treatment, the relative expression level of the *NwNPC2a* gene was significantly reduced (*p* < 0.001) compared to the control, with a reduction of 49.66% (Figure 5).

### 3.6. Predatory Function and Searching Efficiency of N. womersleyi after dsNwNPC2a Interference

**Figure 6:**
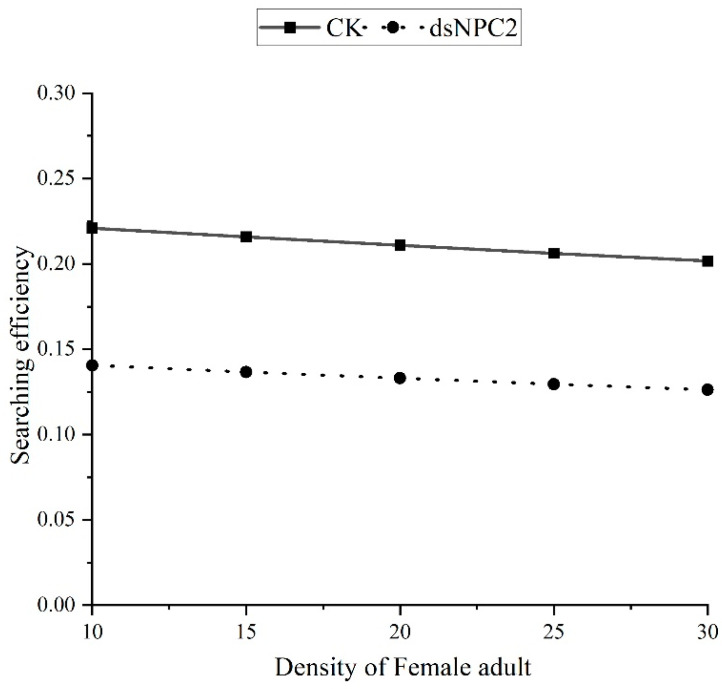
Relationship between the searching efficiency and density of *N. womersleyi* on the female adult state of *T. urticae* treated with RNAi.

The effects of *dsNwNPC2a* interference on the predatory function and searching efficiency of *N. womersleyi* are shown in Table 9 and Table 10 and Figure 6. The results indicate that, compared to the control, the interference-treated *N. womersleyi* exhibited a significantly lower attack coefficient (*a*), handling time (*T_h_*), predatory capacity (*a*/*T_h_*), and maximum predation rate (1/*T_h_*). Additionally, the searching efficiency (*S*) of the interference-treated mites was lower than that of the control and slightly decreased with the increasing prey density.

## 4. Discussion

This study explored the effects of LC_30_ and LC_50_ concentrations of pyridaben on the predatory function and searching efficiency of *N. womersleyi*. Under LC_30_ treatment, an increase was observed in both the predatory capacity (*a*/*T_h_*) of *N. womersleyi* on *T. urticae* larvae and the maximum daily predation rate (1/*T_h_*) on various stages of *T. urticae*. It is speculated that the reduction in handling time (*T_h_*) for *N. womersleyi* when preying on *T. urticae* may lead to less thorough consumption, thereby increasing the number of preys consumed. Compared to previous studies on the predation efficiency of *N. pseudolongispinosus* on *T. urticae* [36], our results indicated that *N. womersleyi* exhibited a Holling Type II functional response to different stages of *T. urticae*. Additionally, it was found that *N. pseudolongispinosus* had a higher searching efficiency for *T. urticae* eggs and larvae compared to nymphs, which aligns with our findings. Research on the predatory functional response of *A. pseudolongispinosus* to *Tetranychus cinnabarinus* [37] found that the functional responses to three different stages of *T. cinnabarinus* also followed Holling Type II. Under similar control treatment densities, the predatory capacity, *a*/*T_h_*, and maximum daily predation rate, *1*/*T_h_*, of *A. pseudolongispinosus* for *T. cinnabarinus* eggs, larvae, and nymphs were lower than those observed in our study for *N. womersleyi* preying on *T. urticae* eggs, larvae, and nymphs. Another study on the effects of sublethal doses of abamectin on the predatory function of *P. persimilis* on *T. urticae* [38] revealed a decrease in the instantaneous attack rate (*a*), an increase in the handling time (*T_h_*), a reduction in the predatory capacity (*a*/*T_h_*), and a decrease in the maximum daily predation rate (1/*T_h_*). This ultimately reduced the control efficiency of *P. persimilis* on the prey. Our study similarly concluded that LC_30_ and LC_50_ concentrations of pyridaben negatively affected the predation efficiency and searching ability of *N. womersleyi*.

The study successfully cloned a NPC2 protein gene, *NwNPC2a*, from *N. womersleyi* for the first time. Under LC_30_ and LC_50_ treatments of pyridaben, the relative expression levels of the *NwNPC2a* gene were found to decrease compared to the control group. As the concentration of pyridaben increased, the relative expression levels of the genes further decreased, indicating that this gene might be involved in the olfactory-related predatory behavior of *N. womersleyi*. The identification of NPC2 proteins from the antennae of the Japanese carpenter ant demonstrated their ability to deliver various hydrophobic signaling substances to chemosensory neurons, marking the first study on the role of NPC2 proteins in olfactory communication [39]. According to Li et al. [21], three full-length *NPC2* genes (*NbNPC2-1, NbNPC2-2*, and *NbNPC2-3*) were cloned based on the transcriptome data of *N. barkeri*, suggesting that NPC2 proteins might be involved in the chemical communication of male mites.

Previous research on the factors affecting RNAi efficiency showed that RNAi efficiency might vary with insect species, dsRNA molecule length, target gene, and other experimental factors [40]. Due to the small size and fast crawling speed of *N. womersleyi*, methods such as microinjection are highly challenging. In RNAi studies on the *Metaseiulus occidentalis* and *N. barkeri*, the feeding method was used to deliver dsRNA into predatory mites. Using the feeding method, RNAi treatment of *NbarNPC2a* and *NbarNPC2b* genes in *N. barkeri* resulted in expression reductions of 59% and 44%, respectively, achieving high levels of gene silencing [32,41]. In this study, the feeding method was used for interference treatment, and the relative expression level of the *NwNPC2a* gene was significantly reduced compared to the control (*p* < 0.001). This indicates that the feeding method effectively interfered with the gene expression in *N. womersleyi*, significantly silencing the target *NPC2* gene. After interference treatment, *N. womersleyi* females showed significant reductions in the attack coefficient (*a*), handling time (*T_h_*), predatory capacity (*a*/*T_h_*), and maximum daily predation rate (*1*/*T_h_*) when preying on *T. urticae*. The searching efficiency (*S*) was also lower than the control and slightly decreased with the increasing prey density. This suggests that NPC2 proteins play an important role in the predation of *T. urticae* by *N. womersleyi*. The internal molecular mechanism by which pyridaben affects the predation ability of *N. womersleyi* may be through the inhibition of *NwNPC2a* expression. Similar findings were reported in RNAi studies, where the daily predation rates of *N. barkeri* significantly decreased under 16 °C and 24 °C conditions after interference with *NbarNPC2a* and *NbarNPC2b* genes [41]. Additionally, RNAi treatment in *P. persimilis* resulted in reduced sensitivity to plant odors, suggesting the involvement of the *PpNPC2a* gene in the recognition of plant volatiles [25]. In the two-spotted spider mite, silencing of a highly expressed *NPC2* transcript (*NP1*) by 48–59% led to reduced feeding and reproduction but did not affect the host-seeking ability. Conversely, downregulation of the *NP5* transcript by more than 50% significantly impaired the host-seeking ability, indicating that *NP1* might be involved in short-range host recognition signals, while *NP5* might be involved in long-range host recognition signals [20,42].

*N. womersleyi* is a widely distributed predatory mite with effective control over various harmful agricultural spider mites. Exploring the effects of pyridaben on *N. womersleyi* and its impact on predatory behavior mechanisms provides theoretical guidance for the field application of pyridaben. In arthropods, NPC2 proteins primarily function as olfactory-binding proteins. Predatory mites may rely on NPC2 proteins to collect HIPVs and other odor molecules to locate and pursue their prey. Further in-depth research is needed to elucidate the relationship between NPC2 proteins and HIPVs within predatory mites and their connection to predatory functions and searching efficiency.

## 5. Conclusions

This study elucidated that pyridaben significantly diminished the predatory efficiency and searching behavior of the predatory mite *N. womersleyi*. The *NPC2* genes played a significant role in the predation of *T. urticae* by *N. womersleyi*. The internal molecular mechanism by which pyridaben affects the predation ability of *N. womersleyi* may involve the inhibition of *NwNPC2a* expression in *N. womersleyi*.

## Figures and Tables

**Table 1:** Primers for PCR.

Gene Name	Sequence (5′-3′)
*NwNPC2a*-F	AATATCTAGTGCTTTTCTGTCTCCT
*NwNPC2a*-R	CTATATTCATATCGATGTCCACGCA

**Table 2:** Primers for qPCR.

Gene Name	Sequence (5′-3′)
*NwNPC2a*-F	TGTCCCGATGCTCTGAAACC
*NwNPC2a*-R	CGTCCAGCAAATGAGCCTTAA
*ACTB*-F	TACGACCAGAAGCGTACAGC
*ACTB*-R	CCAACCGTGAAAAGATGACC

**Table 3:** Primers for dsRNA.

Primer Name	Sequence (5′-3′)
ds*NwNPC2a-F*	TAATACGACTCACTATAGGGCAGCACAGTTCGCTACAGGA
ds*NwNPC2a-R*	TAATACGACTCACTATAGGGTATCGATGTCCACGCAGAAG
ds*GFP-F*	TAATACGACTCACTATAGGGGCCCGAAGGTTATGTACAGG
ds*GFP-R*	TAATACGACTCACTATAGGGCTTTTCGTTGGGATCTTTCG

Note: Underline represents the T7 promoter sequence.

**Table 4:** Bioinformatics analysis websites of *NwNPC2a*.

Analysis Contents	Website Address
Sequence alignment	https://blast.ncbi.nlm.nih.gov/Blast.cgi?PROGRAM=blastn&PAGE_TYPE=BlastSearch&LINK_LOC=blasthome (accessed on 15 August 2023)
Coding protein prediction	https://web.expasy.org/protparam/ (accessed on 15 August 2023)
Conservative domain prediction	http://www.ncbi.nlm.nih.gov/Structure/cdd/wrpsb.cgi (accessed on 15 August 2023)

**Table 5:** The toxicity of pyridaben to females of *Neoseiulus womersleyi*.

Drug Name	Toxicity Regression Equation	*x*^2^/df	LC_30_ (μg/mL)	LC_50_ (μg/mL)
			95% Confidence Interval	95% Confidence Interval
97% Pyridaben	y = −4.455 + 2.833x	6.169/13	24.417	37.395
			20.785~27.956	32.985~42.121

Note: *x*^2^ *=* 6.169 < *x*^2^ (0.05, 13) = 22.363, and the difference was not significant, indicating that the toxicity regression equation can better reflect the relationship between the concentration of the drug and the fatality rate. Toxicity Regression Equation, Y in the table represents the probit value, and X represents the log10 of concentration.

**Table 6:** Predatory function of *N. womersleyi* treated with LC_30_ and LC_50_ concentrations of pyridaben.

Prey Stages	Treatments	*a*	*T_h_* (d)	*a*/*T_h_*	1/*T_h_*	Functional Response Equation	R^2^
Egg	CK	1.152 ± 0.392 a	0.021 ± 0.019 a	54.857	47.620	*Na* = 1.152*N*_0_/(1 + 0.024*N*_0_)	0.977
	LC_30_	0.691 ± 0.206 b	0.029 ± 0.020 a	23.828	34.483	*Na* = 0.691*N*_0_/(1 + 0.020*N*_0_)	0.993
	LC_50_	0.289 ± 0.011 c	0.016 ± 0.005 a	18.063	62.500	*Na* = 0.289*N*_0_/(1 + 0.005*N*_0_)	0.862
Larva	CK	1.068 ± 0.185 a	0.020 ± 0.008 a	53.400	50.000	*Na* = 1.068*N*_0_/(1 + 0.021*N*_0_)	0.833
	LC_30_	0.806 ± 0.153 b	0.013 ± 0.011 a	62.000	76.923	*Na* = 0.806*N*_0_/(1 + 0.010*N*_0_)	0.996
	LC_50_	0.490 ± 0.147 c	0.026 ± 0.014 a	18.846	38.462	*Na* = 0.490*N*_0_/(1 + 0.013*N*_0_)	0.700
Nymph	CK	1.134 ± 0.315 a	0.033 ± 0.012 a	34.364	30.303	*Na* = 1.134*N*_0_/(1 + 0.037*N*_0_)	0.907
	LC_30_	0.547 ± 0.301 b	0.025 ± 0.010 a	22.960	40.000	*Na* = 0.547*N*_0_/(1 + 0.014*N*_0_)	0.943
	LC_50_	0.297 ± 0.356 b	0.027 ± 0.017 a	11.000	37.037	*Na* = 0.297*N*_0_/(1 + 0.008*N*_0_)	0.975
Female adult	CK	0.160 ± 0.133 a	0.031 ± 0.025 b	5.161	32.258	*Na* = 0.160*N*_0_/(1 + 0.005*N*_0_)	0.976
	LC_30_	0.141 ± 0.102 a	0.086 ± 0.061 b	1.640	11.628	*Na* = 0.141*N*_0_/(1 + 0.012*N*_0_)	0.945
	LC_50_	0.193 ± 0.134 a	0.521 ± 0.273 a	0.370	1.919	*Na* = 0.193*N*_0_/(1 + 0.101*N*_0_)	0.842

Note: Attack coefficient/rate, *a*; handling time, *Th*; predation efficiency, *a/Th*; maximum predation rate, 1/*Th*; R is the correlation coefficient. Data in the table are mean ± SE. Different lowercase letters in the table represent the same prey stage with significant differences between the different treatments by Duncan’s new multiple ranges test (*p* < 0.05).

**Table 7:** Searching efficiency of *N. womersleyi* treated with LC_30_ and LC_50_ concentrations of pyridaben.

Prey Stages	Treatments	*a*	*T_h_* (d)	Searching Efficiency Equation
Egg	CK	1.152 ± 0.392 a	0.021 ± 0.019 a	*S* =1.152/(1 + 0.024*N*_0_)
	LC_30_	0.691 ± 0.206 b	0.029 ± 0.020 a	*S* =0.691/(1 + 0.020*N*_0_)
	LC_50_	0.289 ± 0.011 c	0.016 ± 0.005 a	*S* =0.289/(1 + 0.005*N*_0_)
Larva	CK	1.068 ± 0.185 a	0.020 ± 0.008 a	*S* =1.068/(1 + 0.021*N*_0_)
	LC_30_	0.806 ± 0.153 b	0.013 ± 0.011 a	*S* =0.806/(1 + 0.010*N*_0_)
	LC_50_	0.490 ± 0.147 c	0.026 ± 0.014 a	*S* =0.490/(1 + 0.013*N*_0_)
Nymph	CK	1.134 ± 0.315 a	0.033 ± 0.012 a	*S* =1.134/(1 + 0.037*N*_0_)
	LC_30_	0.547 ± 0.301 b	0.025 ± 0.010 a	*S* =0.547/(1 + 0.014*N*_0_)
	LC_50_	0.297 ± 0.356 b	0.027 ± 0.017 a	*S* =0.297/(1 + 0.008*N*_0_)
Female adult	CK	0.160 ± 0.133 a	0.031 ± 0.025 b	*S* = 0.160/(1 + 0.005*N*_0_)
	LC_30_	0.141 ± 0.102 a	0.086 ± 0.061 b	*S* = 0.141/(1 + 0.012*N*_0_)
	LC_50_	0.193 ± 0.134 a	0.521 ± 0.273 a	*S* = 0.193/(1 + 0.101*N*_0_)

Note: Attack coefficient/rate, *a*; handling time, *Th*. Data in the table are mean ± SE. Different lowercase letters in the table represent the same prey stage with significant differences between the different treatments by Duncan’s new multiple ranges test (*p* < 0.05).

**Table 8:** Full-length sequence analysis results of *NwNPC2a*.

Gene Name	GenBank Accession Number	Sequence Length	Amino Acid Length	Relative Molecular Mass	Theoretical PI
*NwNPC2a*	OR897818.1	549 bp	182	46.03 kDa	5.03

**Table 9:** Predatory function of female adult *N. womersleyi* treated with RNAi.

Treatments	*a*	*T_h_* (d)	*a*/*T_h_*	1/*T_h_*	Functional Response Equation	R^2^
CK	0.232 ± 0.076 *	0.023 ± 0.007	10.087	43.478	*Na* = 0.232*N*_0_/(1 + 0.005*N*_0_)	0.868
*dsNPC2*	0.149 ± 0.068	0.040 ± 0.013 *	3.725	25.000	*Na* = 0.149*N*_0_/(1 + 0.006*N*_0_)	0.909

Note: Attack coefficient/rate, *a*; handling time, *T_h_*; predation efficiency, *a*/*T_h_*; maximum predation rate, 1/*T_h_*; R is the correlation coefficient. Data in the table are mean ± SE. Significant differences were compared and plotted using the independent samples *t*-test. * Indicates *p* < 0.05.

**Table 10:** Searching efficiency of female adult *N. womersleyi* treated with RNAi.

Treatments	*a*	*T_h_* (d)	Searching Efficiency Equation
CK	0.232 ± 0.076 *	0.023 ± 0.007	*S* = 0.232/(1 + 0.005*N*_0_)
*dsNPC2*	0.149 ± 0.068	0.040 ± 0.013 *	*S* = 0.149/(1 + 0.006*N*_0_)

Note: Attack coefficient/rate, *a*; handling time, *T_h_*; R is the correlation coefficient. Data in the table are mean ± SE. Significant differences were compared and plotted using the independent samples *t*-test. * Indicates *p* < 0.05.

## Data Availability

The data presented in this study are available upon request from the corresponding author.
